# Application of laparoscopic distal pancreatectomy for normal anatomy after hiatal hernia repair: A case report

**DOI:** 10.1002/ccr3.5832

**Published:** 2022-05-12

**Authors:** Akira Yoneda, Shunsuke Murakami, Hanako Tetsuo, Saeko Fukui, Takayuki Miyoshi, Tatsuya Okamoto, Amane Kitasato, Hiroaki Takeshita, Tamotsu Kuroki

**Affiliations:** ^1^ Department of Surgery National Hospital Organization Nagasaki Medical Center Omura Japan

**Keywords:** distal pancreatectomy, hiatal hernia, pancreatic tumor

## Abstract

We describe a case of pancreatic tumor associated with a giant type IV hiatal hernia that had prolapsed into the posterior mediastinum. Hiatal hernia repair should be performed first because it enables laparoscopic distal pancreatectomy to be performed in the normal anatomical position.

## Abstract 2

In cases requiring pancreatic tumor surgery associated with a giant type IV hiatal hernia, hernia repair should be initially performed to ensure that pancreatectomy can be performed safely and in the normal anatomical position.

## 1 INTRODUCTION

Acquired hiatal hernia is classified according to the location of the gastroesophageal junction and the extent of the hernia. In type III (mixed) hiatal hernia, more than 30% of the stomach is herniated with the gastroesophageal junction.^1^ In type IV giant hernias (0.3%) and congenital hernias (0.2%), organs other than the stomach are herniated. The most frequently herniated organs include the colon, small intestine, omentum, and spleen.^2^, ^3^ Intra‐abdominal organs, such as the transverse colon, can herniate along with the stomach; however, herniation of the pancreas is very rare.

In simultaneous hiatal hernia repair and distal pancreatectomy, understanding the anatomical features is difficult because of the displacement of the pancreas. Moreover, thus far, no studies have reported cases of neoplastic lesions in a prolapsed pancreas.

We present the case of an elderly patient with a pancreatic tumor associated with a giant type IV hernia that had prolapsed into the posterior mediastinum and required resection. We also present a literature review on surgical treatment of this rare disease.

## 2 CASE PRESENTATION

**FIGURE 1:**
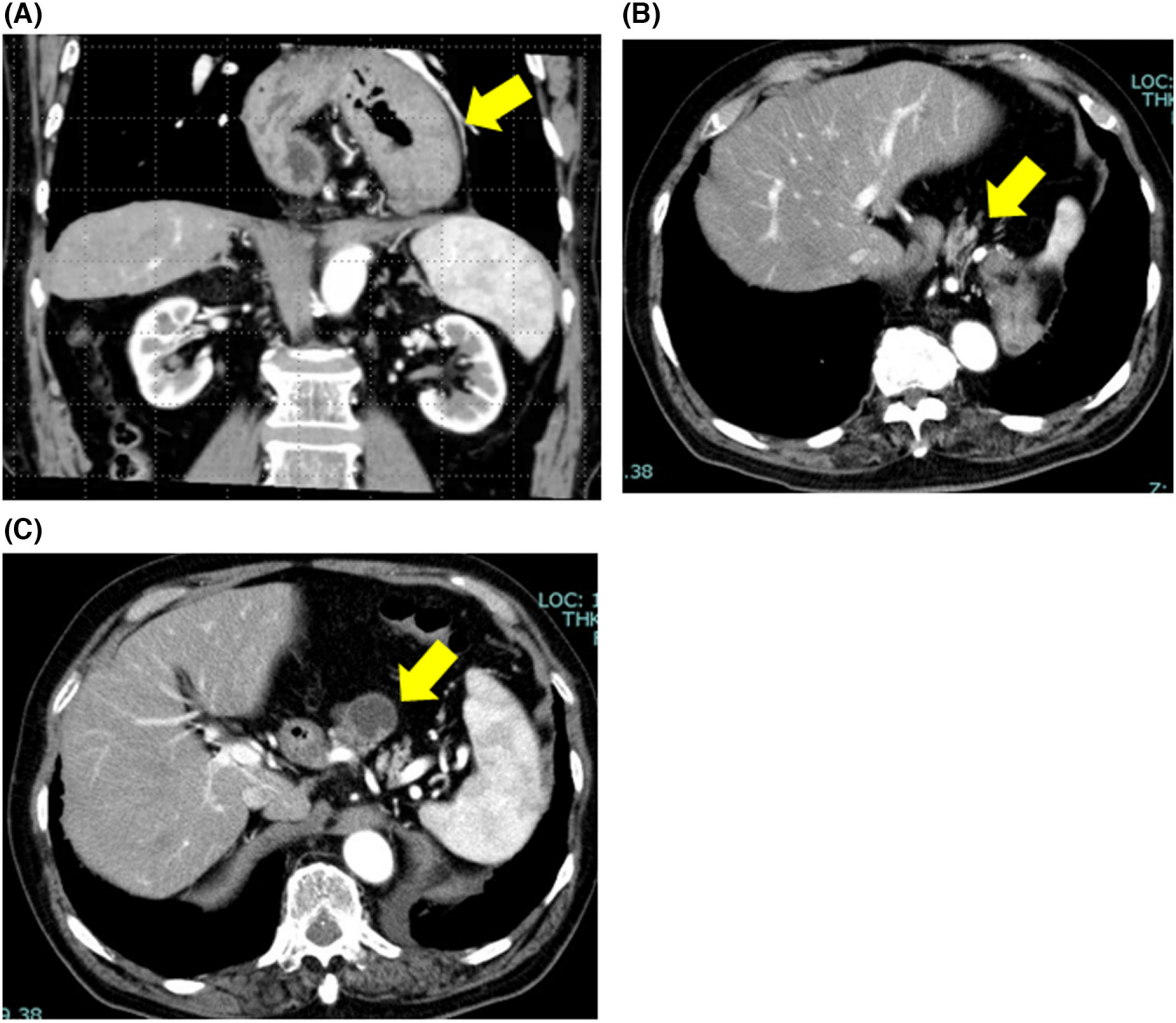
Computed tomography images showing (A) prolapse of the stomach, (B) prolapse of the pancreatic body, and (C) an 8‐mm solid component in the pancreatic body

**FIGURE 2:**
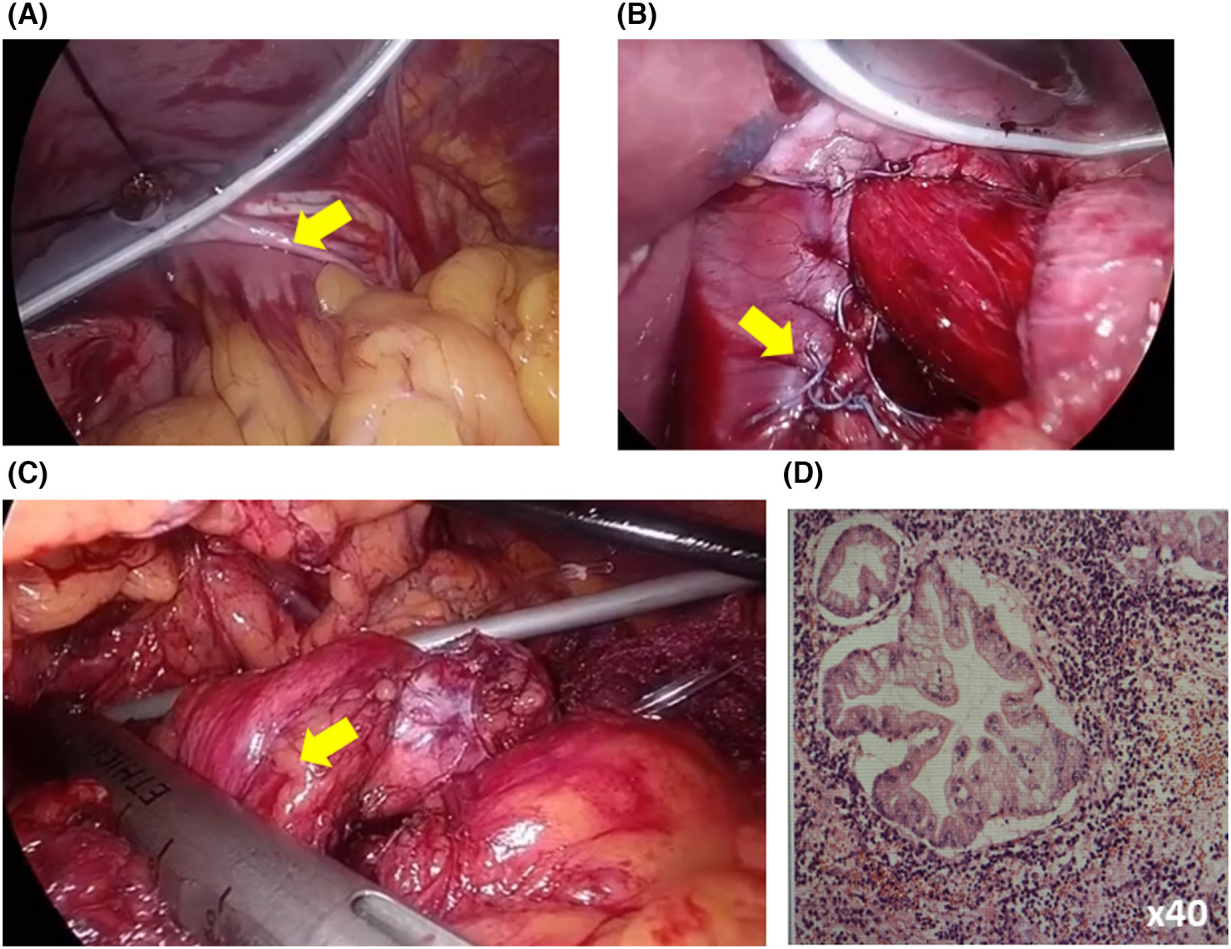
Laparoscopic images showing (A) a huge hiatal hernia, wherein a large part of the stomach had prolapsed into the mediastinum, (B) suturing of the hernia orifice with a nonabsorbable thread, and (C) distal pancreatectomy in the normal position. (D) Histopathological findings were intraductal papillary mucinous carcinoma

An 85‐year‐old man with dysphagia was admitted to our hospital. Computed tomography (CT) revealed prolapse of a massive hiatal hernia involving the stomach (Figure 1A) and pancreatic body (Figure 1B). His CT findings also showed an 8‐mm enhanced solid component in the cyst, which was found in the pancreas body (Figure 1C). We performed UGIS preoperatively, which revealed findings of herniation of the entire stomach into the mediastinum. We suspected that an intraductal papillary mucinous neoplasm was the most likely diagnosis, and surgery was indicated. Hiatal hernia repair was performed, followed by laparoscopic distal pancreatectomy. Although the patient was 85 years old, we determined that he would be able to tolerate the simultaneous surgery because of his good performance status and cardiopulmonary function. Ports were placed in the umbilical region, bilateral hypochondria, and bilateral upper abdomen. A large hiatal hernia was also observed. A large part of the stomach had prolapsed into the mediastinum (Figure 2A). The adhesion between the hernia sac and the omentum was peeled off. The hernia orifice was sutured with a nonabsorbable thread while pulling the esophagogastric junction (Figure 2B). Fundoplication was performed from the front, and hiatal hernia repair was completed. The pancreas then returned to its normal position. Tunneling of the pancreatic parenchyma was performed immediately above the portal vein. The pancreatic parenchyma was dissected using a laparoscopic linear stapler (Figure 2C). Splenic arteries and veins were dissected. The pancreatic tail and spleen were detached from the retroperitoneum, and the specimen was removed. In this case, gastric stasis occurred but improved conservatively. Esophageal reflux was not observed, and the patient was transferred to another hospital on postoperative day 13. Histopathological findings were intraductal papillary mucinous carcinoma (Figure 2D).

## 3 DISCUSSION

Hiatal hernia may be transient or permanent and causes the stomach to pass through the diaphragm and enter the chest. The operative principles of a hernia repair are the same for laparoscopic and open approaches. They involve the dissection of the sac and reduction in the hernia followed by complete excision of the sac from the mediastinum. After the esophagus is mobilized, the hiatal defect is closed by interrupted nonabsorbable sutures. Gastric fixation, such as anterior gastropexy, may safely be used in addition to hiatal repair to prevent recurrence.^4^ Surgical intervention, including hernia reduction, closure of the esophageal hiatus, and anti‐reflux procedure, may be necessary for types II, III, and IV hernias with severe esophagitis. It may also involve other abdominal organs, as observed in the present case. The most common organs associated with the stomach are the colon, small intestine, and omentum. A giant hiatal hernia has been reported frequently; however, cases of giant hernia with pancreatic prolapse are extremely rare.^5^ Stretching of the transverse mesocolon has been reported to increase the mobility of the pancreas owing to greater relaxation of the posterior adherent fascia. A recent study of a human cadaver reported a posterior pancreatic fascia covering the posterior surface of the main body of pancreas but was unable to locate the anterior renal fascia, possibly because of age‐related degeneration of the adrenal glands.^6^ In the elderly, the pancreas may be more mobile owing to connective tissue degeneration, making it more likely to migrate from a hiatal hernia. Pancreatic hernias are asymptomatic,^7^, ^8^ and most cases are discovered incidentally during CT scans performed to evaluate features characteristic of giant esophageal hernias, such as abdominal pain,^9^, ^10^ vomiting,^11^, ^12^ dysphagia,^3^ and dyspnea.^13^ In this case, the patient complained of dysphagia and was found to have a giant hiatal hernia with pancreatic prolapse. Herniation of pancreas can be found as a sequel to acute pancreatitis. Acute pancreatitis as a sequela of this mechanism is very rare.^14^ However, there are no reports of neoplastic lesions in the prolapsed pancreas. In this case, the pancreas with a tumor requiring resection had prolapsed into the posterior mediastinum due to a giant hiatal hernia. We performed hiatal hernia surgery before pancreatic resection to ensure that a safe pancreatic resection is conducted. Preceding hiatal hernia repair allows pancreatic resection to be performed in the normal anatomical position, thereby increasing safety of the procedure. One study reported a case of pancreatitis and bile duct dilatation secondary to a giant hiatal hernia of the pancreatic tail.^14^ Therefore, pancreatic resection without esophageal hiatal hernia repair in the present case may pose a risk of inflammation extending into the mediastinum when complications, such as pancreatic fistula, develop.

## 4 CONCLUSION

In simultaneous hiatal hernia repair and distal pancreatectomy, understanding the anatomy is difficult because of the displacement of the pancreas. Therefore, hiatal hernia repair should be performed first because it enables laparoscopic distal pancreatectomy to be performed in the normal anatomical position.

## AUTHOR CONTRIBUTION

Akira Yoneda, Shunsuke Murakami, Hanako Tetsuo, Saeko Fukui, Takayuki Miyoshi, Tatsuya Okamoto, Amane Kitasato, Hiroaki Takeshita, and Tamotsu Kuroki involved in manuscript writing and literature review. All authors have read and approved the final manuscript.

## CONFLICT OF INTEREST

The authors have no conflict of interest to declare.

### ETHICAL APPROVAL

This case study was approved by the Biomedical Ethics Committee.

### CONSENT

Written informed consent was obtained from the patient for publication of this case report and any accompanying images. This study was approved by the Ethics Committee of National Hospital Organization Nagasaki Medical Center.

## Data Availability

All data collected during this study are available from the corresponding author.
